# Understanding P wave pathology and other highlights

**DOI:** 10.1111/anec.13063

**Published:** 2023-05-18

**Authors:** Mark Haigney

**Affiliations:** ^1^ Hebert School of Medicine Bethesda MD USA

In this issue of the Annals, ISHNE members Professors Bayes de Luna and Bacharova substantially contribute to our understanding of atrial pathology as revealed in the electrocardiographic P wave. In so doing, the extend the work that Professor Bayes de Luna began nearly 40 years ago.

In this remarkable paper, they taxonomize the three patterns of typical advanced inter‐atrial block (A‐IAB), as well as describe atypical patterns (i.e. with P wave durations less than 120 ms but positive–negative polarity in the inferior leads), explain the differentiation between A‐IAB and atrial enlargement, and make the case that A‐IAB may be as important a predictor of future stroke as atrial fibrillation.

For those unfamiliar with the phenomenon that has been dubbed “Bayes Syndrome” (the Professor modestly avoids this eponym), this paper is the perfect introduction.

In other important developments at Annals, we have added two important individuals to our masthead.

Professor Linda Johnson, MD, PhD Associate Professor, Cardiovascular Epidemiology, Lund University has joined us in the role of Associate Editor. Her work combines a profound understanding of cardiovascular epidemiology with an interest in ambulatory ECG monitoring for assessment of atrial fibrillation risk and autonomic dysfunction. I am very grateful that she has accepted our invitation and look to encourage submissions in her areas of expertise.

In a first for the Annals, we welcome our new Social Media Editor, Andrés Felipe Miranda‐Arboleda, MD. Andres hails from Medellín, Colombia, but is currently finishing his electrophysiology fellowship at Queen's University, Kingston, Ontario. Please be sure to look out for his posts on Twitter, WeChat, and other platforms to keep you informed about important new Annals publications as well as throw‐backs to classic papers that have stood the test of time.

Welcome aboard, Linda and Andres!

